# RCAF for patient-level thyroid ultrasound malignancy prediction under leakage-free evaluation and calibration

**DOI:** 10.1038/s41598-026-61342-8

**Published:** 2026-07-16

**Authors:** Mennatallah Sherif, Eman K. Elsayed, Mohanad A. Deif

**Affiliations:** 1 Department of Computer Science, College of Information Technology, Misr University for Science and Technology (MUST), P.O. Box 77, Giza, Egypt; 2 College of Information Technology, Misr University for Science and Technology (MUST), P.O. Box 77, Giza, Egypt

**Keywords:** Thyroid ultrasound, Patient-level classification, Thyroid nodule malignancy prediction, Multiple instance learning, Region-aware fusion, Probability calibration, Deep learning, Cancer, Computational biology and bioinformatics, Health care, Medical research, Oncology

## Abstract

Accurate differentiation between benign and malignant thyroid nodules on ultrasound remains clinically important, yet interpretation is operator-dependent and subject to inter-observer variability. Recent deep learning studies report strong performance for thyroid ultrasound classification, but many prior approaches remain centred on image-level prediction, with limited emphasis on patient-level baselines, calibration-aware evaluation, and mask-related shortcut analysis. To address these gaps, we present a patient-level thyroid ultrasound malignancy prediction framework centred on a Region-Aware Context-Aware Fusion (RCAF) model evaluated under a strict leakage-free protocol. RCAF combines lesion-focused and context-preserving frame representations through a dual-branch design with gated fusion, followed by attention-based multiple instance learning (AttnMIL) for patient-level aggregation. The framework incorporates development-only probability calibration and threshold selection before single-shot evaluation on an untouched independent test cohort. Experiments on the public ThyroidXL benchmark show that RCAF outperforms stronger fair patient-level baselines, including image-only, transformer-based, and lesion-only comparators. Calibration analysis improves probability reliability, threshold analysis demonstrates stable behaviour under clinically relevant operating conditions, and shortcut sensitivity experiments show that naive mask concatenation produces shortcut-prone gains, whereas RCAF degrades by only 0.001 ROC-AUC under within-patient mask permutation, supporting principled region-aware reasoning. For cross-domain assessment, RCAF was evaluated on TN5000, a Chinese thyroid ultrasound dataset acquired under different imaging conditions; following domain adaptation and 8-view test-time augmentation, the model achieved AUC = 0.914 [0.879–0.948] on a class-balanced validation subset. Overall, RCAF constitutes a strong patient-level thyroid ultrasound classification framework, with encouraging cross-domain adaptability. Broader prospective multi-centre validation remains necessary before clinical deployment.

## Introduction

Thyroid nodules represent one of the most common endocrine findings encountered in clinical imaging practice, and ultrasound is routinely employed as the primary assessment tool owing to its non-invasive nature, broad availability, and cost efficiency. However, ultrasound interpretation is inherently operator-dependent and can be affected by lesion appearance variability, acquisition conditions, and inter-observer disagreement. These challenges have motivated growing interest in artificial intelligence systems for thyroid ultrasound analysis, particularly for benign–malignant discrimination and support of downstream management decisions. Large multicenter studies have already shown that deep learning can provide clinically meaningful assistance in thyroid nodule diagnosis and workflow support^1,2^, while recent prospective evidence confirms that inter-observer variability remains a meaningful challenge in ultrasound-based thyroid nodule risk stratification, reinforcing the need for robust decision-support tools^3^.

Recent deep learning approaches to thyroid ultrasound classification span image-level convolutional classifiers^4,5^, transfer-learning frameworks^6^, visually interpretable systems^7^, and multimodal methods^8,9^. Review studies confirm that the field has expanded rapidly across classification, detection, and segmentation, while also identifying persistent challenges in generalisation, reproducibility, and clinical deployment^10,11^. Despite this progress, translation to patient-level decision support remains incomplete. Recent work on attention-based multiple instance learning (MIL) has begun to address multi-frame aggregation in clinical imaging settings^12,13^, yet patient-level evaluation against strong baselines under leakage-free protocols remains underexplored in thyroid ultrasound specifically.

A related direction has focused on lesion localisation and segmentation-guided learning. This is clinically intuitive because malignancy-associated cues depend strongly on nodule morphology, margins, echogenicity, and surrounding structural context. Segmentation-oriented studies have emphasised the importance of accurate lesion delineation in thyroid ultrasound images^14^. More recently, mask-guided classification frameworks have incorporated segmentation-derived lesion guidance to direct feature extraction toward diagnostically relevant regions^15^. While these studies support the value of lesion-aware modelling, they do not fully resolve how lesion information should be combined with broader contextual evidence at the patient level, nor do they address whether naive image–mask concatenation may introduce shortcut-prone behaviour rather than principled lesion-guided reasoning.

The introduction of ThyroidXL^16^ addressed the long-standing scarcity of large public datasets with pathology-validated labels, providing more than 11,000 expert-annotated images from over 4000 patients and making stronger patient-level experimentation possible. However, the availability of a benchmark alone does not resolve the methodological challenges of fair patient-level baseline design, structured mask usage, or calibration-aware evaluation. In particular, malignancy probabilities may ultimately support referral, biopsy recommendation, or triage decisions, making probability calibration an essential component of reliable deployment^17^—yet calibration-aware patient-level evaluation remains underreported in thyroid ultrasound computer-aided diagnosis. More broadly, real-world generalisation and data quality remain central barriers to trustworthy clinical AI systems^18,19^.

Despite this progress, several methodological gaps remain: many studies operate at the image level rather than the patient level, lesion-guided approaches are rarely evaluated against stronger fair patient-level baselines or tested for shortcut-prone mask behaviour, and calibration-aware evaluation is underreported. Motivated by these gaps, this study proposes the Region-Aware Context-Aware Fusion (RCAF) framework for patient-level thyroid ultrasound malignancy prediction, evaluated under a strict leakage-free protocol with explicit shortcut analysis, probability calibration, and threshold transfer. The remainder of the paper is organised as follows: Sect. “Related work” reviews related work, Sect. “Problem formulation and research gap” formulates the problem and identifies the research gap, Sect. “Materials and methods” details the materials and methods, Section Experimental setup describes the experimental setup, Sect. “Results” reports the results, Section “Discussion” discusses the findings, Sect. “Limitations and future work” presents limitations and future work, and Section “Conclusion” concludes the paper.

## Related work

Recent deep learning approaches to thyroid ultrasound classification fall into four broad lines of work, each of which directly motivates a specific design choice in the proposed framework.

Large-scale clinical studies by Peng et al.^1^ and Ha et al.^2^ established the translational potential of AI-assisted thyroid nodule diagnosis, demonstrating that deep learning can support nodule management at scale across multicenter settings. Both systems operate primarily at the nodule or image level and report strong discrimination performance, but neither addresses patient-level multi-frame aggregation, calibration-aware evaluation, or the distinction between principled lesion guidance and shortcut-prone mask injection—the three methodological gaps that the present work targets directly. In addition, recent prospective work has reaffirmed that inter-observer variability remains a meaningful limitation in ultrasound-based thyroid nodule assessment, further motivating robust and reproducible computer-aided systems^3^.

Image-level convolutional classifiers have formed the dominant line of work in thyroid ultrasound deep learning. Yang et al.^4^ demonstrated the feasibility of ResNet-based thyroid nodule classification with Grad-CAM interpretability, while Rho et al.^5^ extended deep learning to thyroid micronodules, a particularly challenging small-lesion setting. Ajilisa et al.^6^ proposed a multi-level transfer learning strategy using an improved Inception network, and Liu et al.^7^ combined classification with visual interpretability analysis and radiologist comparison. Tao et al.^8^ further explored multimodal B-mode input to improve classification beyond standard grey-scale imaging. More recent studies have continued this trend using alternative transfer-learning and transformer-based classification pipelines^20–22^. Collectively, these studies confirm that deep learning is effective for image-level thyroid classification, but they share a common architectural limitation: frame-level predictions are not aggregated into a patient-level decision, which is the clinically appropriate unit of diagnosis.

Lesion localisation and segmentation-guided learning represent a complementary and clinically motivated direction. Dong et al.^14^ proposed a dual-path attention-enhanced UNet++ for thyroid nodule segmentation, reinforcing the clinical importance of accurate lesion delineation. Complementarily, Yang et al.^23^ studied joint lesion localisation and benign–malignant discrimination, further emphasising the value of spatially informed learning. More recently, Liu et al.^15^ introduced a dual-branch mask-guided attention framework for nodule classification that directs feature extraction toward diagnostically relevant regions. This work is architecturally related to the proposed RCAF model, but differs in two important respects: it operates at the image level rather than the patient level, and it does not examine whether mask-guided inputs can produce shortcut-prone behaviour—a question the present work addresses explicitly through a shuffled-mask diagnostic experiment applied to both the naive mask-channel baseline and the proposed model itself. Segmentation-oriented advances have also continued with more powerful hybrid attention models for thyroid ultrasound target delineation^24^.

Benchmarking and calibration have emerged as increasingly important concerns in thyroid ultrasound AI. The introduction of ThyroidXL^16^ addressed the long-standing absence of large, pathology-validated public benchmarks, providing more than 11,000 expert-annotated images from over 4,000 patients with official train/test splits. Recent review articles have highlighted the broader maturation of thyroid ultrasound AI while also identifying persistent issues in reproducibility, generalisation, and clinical translation^10^. Saini et al.^9^ demonstrated the value of uncertainty-aware multimodal ultrasound classification, and Balanya et al.^17^ reinforced the importance of post-hoc probability calibration for reliable deep neural network deployment^25^. Despite these advances, calibration-aware patient-level evaluation and explicit shortcut analysis remain underreported in thyroid ultrasound AI—the two methodological contributions that this work integrates into a unified leakage-free framework.

## Problem formulation and research gap

### Problem formulation

In this study, thyroid malignancy prediction is formulated as a patient-level classification task under a strict leakage-free evaluation design. Each patient is represented as a bag of ultrasound frames, and all frames belonging to the same examination share a single patient-level diagnostic label, either benign or malignant. The objective is to estimate one malignancy probability for the entire patient rather than producing disconnected frame-level predictions. This formulation is more clinically appropriate because diagnostic decisions are made at the case level, not at the level of individual frames.

### Research gap

Although deep learning for thyroid ultrasound analysis has advanced considerably, several methodological limitations remain common in prior work. First, many studies emphasize image-level prediction even though the clinically relevant task is patient-level diagnosis. Second, patient-level modeling is often addressed using simple pooling rules or with limited comparison against stronger patient-level baselines. Third, while lesion masks can provide useful anatomical guidance, naive mask usage, such as direct image–mask channel concatenation, may lead to shortcut-prone behavior rather than principled lesion-aware reasoning. Finally, calibration quality and threshold transfer are often underreported, despite their practical importance for clinically meaningful decision support.

These limitations motivate the need for a framework that addresses four requirements simultaneously: patient-level modeling rather than isolated frame-wise scoring, stronger and fairer patient-level baseline comparison, structured lesion-guided feature extraction that avoids naive mask shortcut behavior, and leakage-free calibration and threshold selection performed strictly within the development protocol. The importance of rigorous evaluation design and the risk of data leakage in machine-learning-based science have been highlighted as critical barriers to reproducible AI^26^.

### Main contributions

The main contributions of this study are as follows:

1. Thyroid ultrasound malignancy prediction is formulated as a leakage-free patient-level task that integrates multiple frames from the same examination into a single clinically meaningful prediction.
2. The proposed Region-Aware Context-Aware Fusion (RCAF) model combines lesion-focused and context-preserving frame representations through structured gated fusion rather than naive raw mask concatenation.
3. RCAF is evaluated against a stronger set of fair patient-level baselines, including image-only AttnMIL, lesion-only crop + AttnMIL, a transformer-based bag model, and simple aggregation baselines.
4. Shortcut sensitivity analysis is performed for naive mask concatenation to justify structured region-aware integration instead of direct image–mask fusion.
5. Patient-level calibration, reliability assessment, and development-only threshold selection are incorporated into the evaluation pipeline to support clinically meaningful operating-point analysis.
6. External domain-adapted validation is performed on TN5000 to assess cross-domain adaptability under different scanner, acquisition, and population conditions.

## Materials and methods

### Materials

**Fig. 1:**
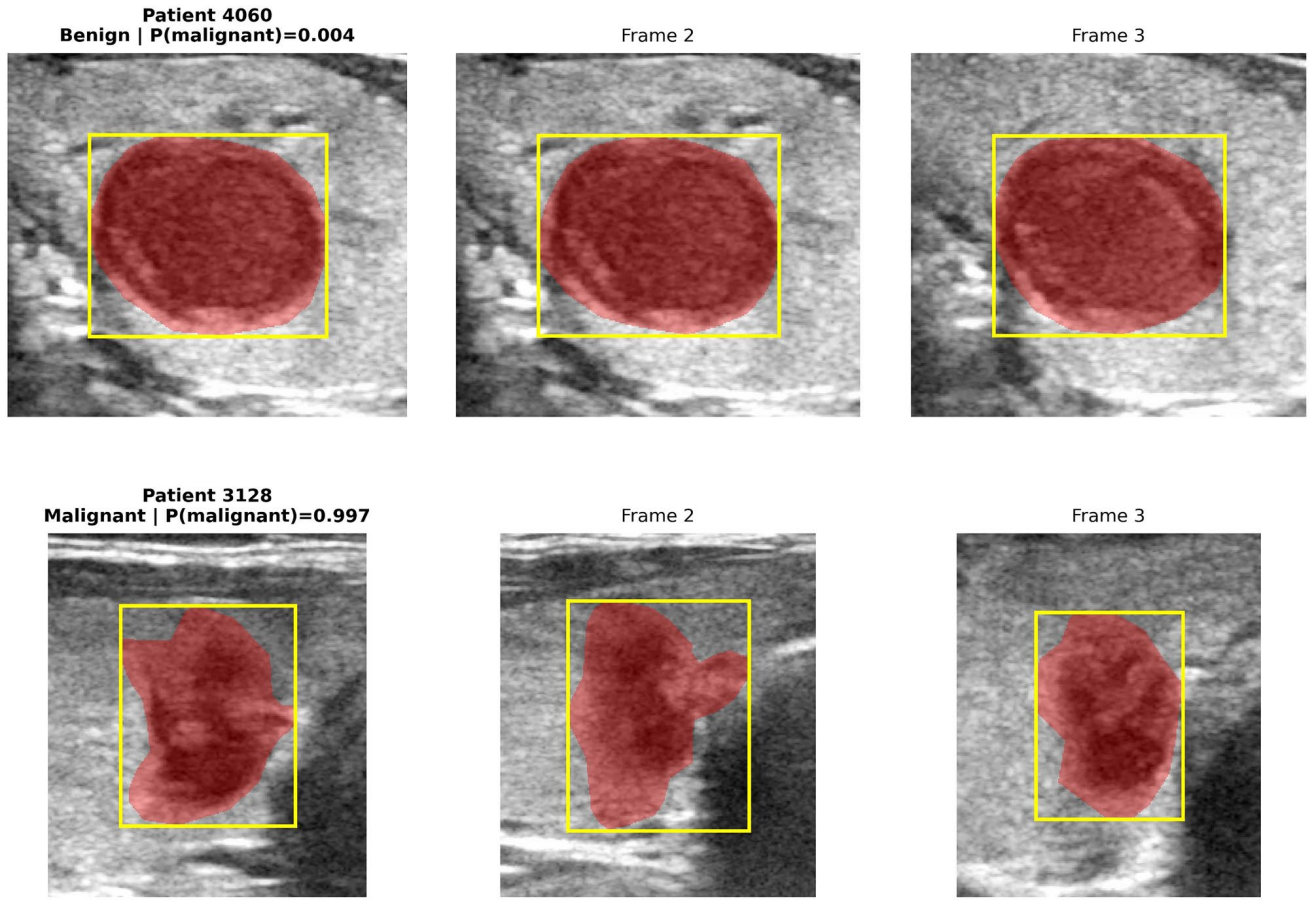
Representative patient-level ultrasound bags from the official test set, illustrating intra-patient variability across frames.

This study uses the public ThyroidXL dataset^16^, a large-scale, pathology-validated benchmark for thyroid ultrasound analysis containing expert-annotated images with patient-level diagnostic labels confirmed by histopathological examination. The dataset is particularly suited to patient-level modelling because each examination contains multiple ultrasound frames acquired during routine clinical practice. After preprocessing and metadata validation, the cohort comprised 4093 patients and 11,635 images, partitioned by the official split into development and independent test cohorts with differing class balance, alongside TIRADS scores and patient demographics. Full dataset, class-distribution, and split statistics are summarised in Table 1, and representative patient-level bags illustrating intra-patient variability across frames are shown in Fig. 1. Ground-truth malignancy labels were derived from histopathological examination, and binary segmentation masks delineating the thyroid nodule region were available for each image. These masks were not treated as standalone prediction targets; instead, they were used as structured anatomical guidance to separate lesion-focused information from surrounding tissue context.

**Table 1:** Summary statistics of the ThyroidXL dataset used in this study.

Statistic	Development set	Test set
Number of patients	3354	739
Number of images	9541	2094
Frames per patient (min–max)	1–10	1–8
Mean frames per patient	2.84	2.83
Median frames per patient	3	3
Benign patients, *n* (%)	2477 (73.9%)	386 (52.2%)
Malignant patients, *n* (%)	877 (26.1%)	353 (47.8%)
TIRADS 1, *n*	24	0
TIRADS 2, *n*	328	45
TIRADS 3, *n*	1090	182
TIRADS 4, *n*	1024	215
TIRADS 5, *n*	888	297
Mean age, years	$48.1 \pm 12.7$ (range 12–94)	
Female, *n* (%)	3650 (89.2%)	

### Methods

**Fig. 2:**
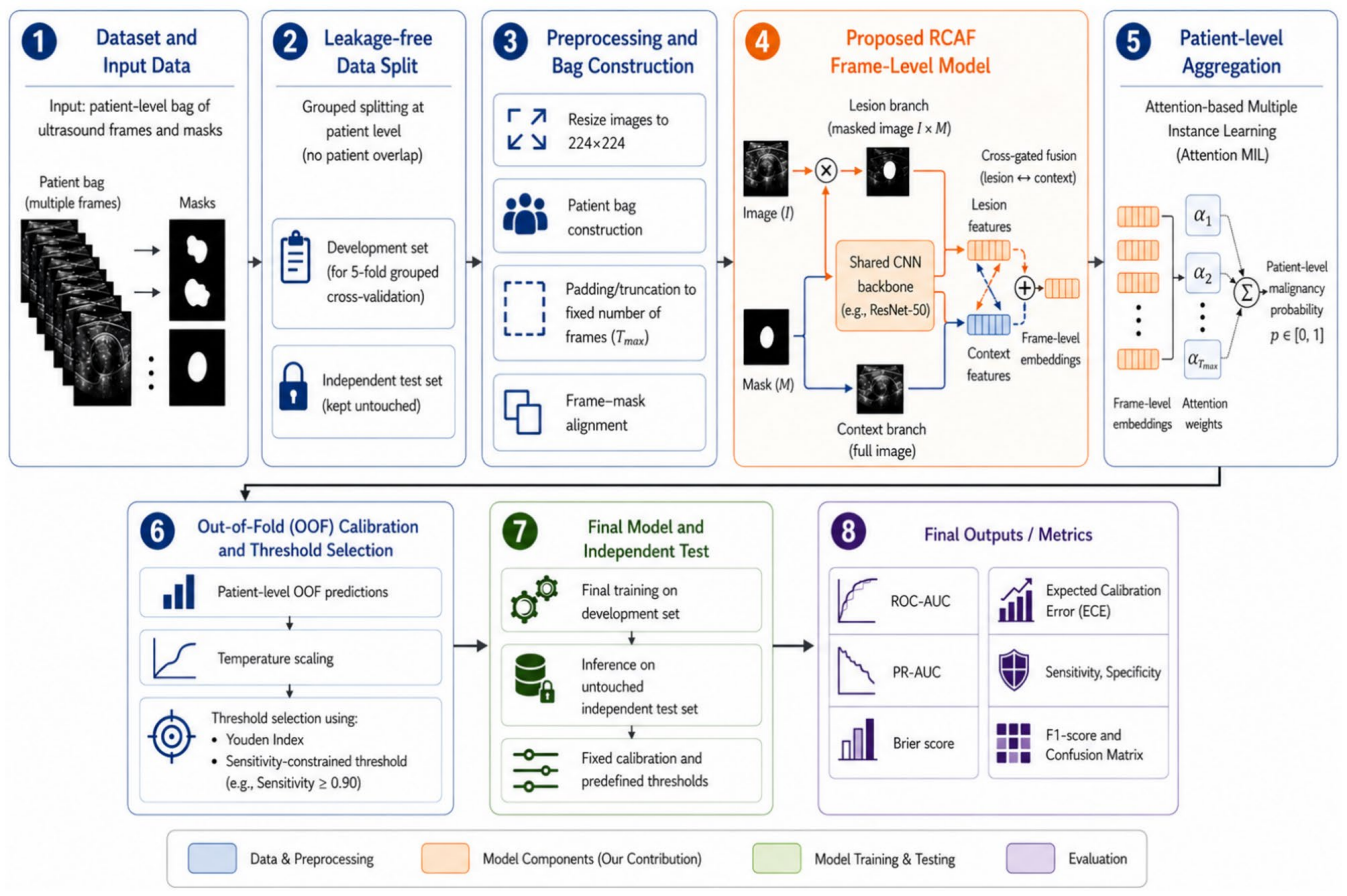
Overview of the proposed patient-level thyroid ultrasound classification pipeline.

This section describes the proposed patient-level thyroid ultrasound classification framework, centred on the Region-Aware Context-Aware Fusion (RCAF) model and evaluated under a strict leakage-free patient-level protocol. An overview of the framework is shown in Fig. 2. Starting from raw ultrasound frames and their corresponding segmentation masks, the pipeline proceeds through a leakage-free patient-level split, preprocessing and patient-bag construction, frame-level region-aware encoding with the proposed RCAF model, patient-level aggregation using attention-based multiple instance learning, development-only out-of-fold calibration and threshold selection, and final single-shot evaluation on the official independent test cohort.

The following subsections detail each methodological component of the proposed framework in order, corresponding to the pipeline stages illustrated in Fig. 2.

#### Leakage-free data split

A strict patient-level partitioning protocol was adopted to prevent information leakage. The official independent test cohort was kept fully isolated and used only once for final model evaluation. The remaining patients formed the development cohort, within which grouped stratified five-fold cross-validation was performed at the patient level, so that all frames from a given patient remained within a single fold and never appeared across both training and validation subsets. This grouped design was essential because image-level splitting would risk inflated performance by allowing patient-specific visual characteristics to leak across subsets. The same partitioning strategy was used across the proposed model and all fair baseline models to ensure unbiased comparison and reliable estimation of patient-level generalisation.

#### Preprocessing and bag construction

All ultrasound images were resized to a fixed spatial resolution of $224 \times 224$ pixels, and pixel intensities were normalised to the range [0, 1] to facilitate stable optimisation. Segmentation masks were resized using nearest-neighbour interpolation to preserve their binary structure. During training, data augmentation comprising random horizontal flipping, small geometric perturbations, and mild intensity variation was applied to improve generalisation and reduce sensitivity to acquisition variability. These procedures were applied consistently across the proposed method and all fair baseline models.

To formalise the patient-level learning setting, each examination was represented as a bag of ultrasound frames associated with a single patient-level diagnostic label. For each patient *i*, the input was represented as

1 $$\begin{aligned} \mathcal {B}_i = \{x_{i1}, x_{i2}, \dots , x_{iT_i}\}, \end{aligned}$$

where $T_i$ denotes the number of ultrasound frames acquired for patient *i*, and all frames within the same bag (Eq. (1)) share the same patient-level label $y_i \in \{0,1\}$, where 0 denotes benign and 1 denotes malignant.

To enable efficient patient-level learning with a fixed tensor structure, the number of frames per patient was standardised to a maximum of $T_{\max } = 10$. Because the dataset maximum coincides with $T_{\max }$, no patient examination was truncated in this study; all patients had at most 10 frames and are therefore fully represented in their bag. Patients with fewer than $T_{\max }$ frames were padded with zero-valued frames, and a binary frame-validity mask was used to exclude padded entries from attention weight computation during both training and inference. Should the framework be applied to datasets where examinations routinely exceed 10 frames, a principled truncation policy such as retaining the $T_{\max }$ frames with the highest individual frame-level malignancy scores from a lightweight pre-filter would be necessary, and its effect on patient-level performance should be validated explicitly.

#### Proposed RCAF frame-level model

**Fig. 3:**
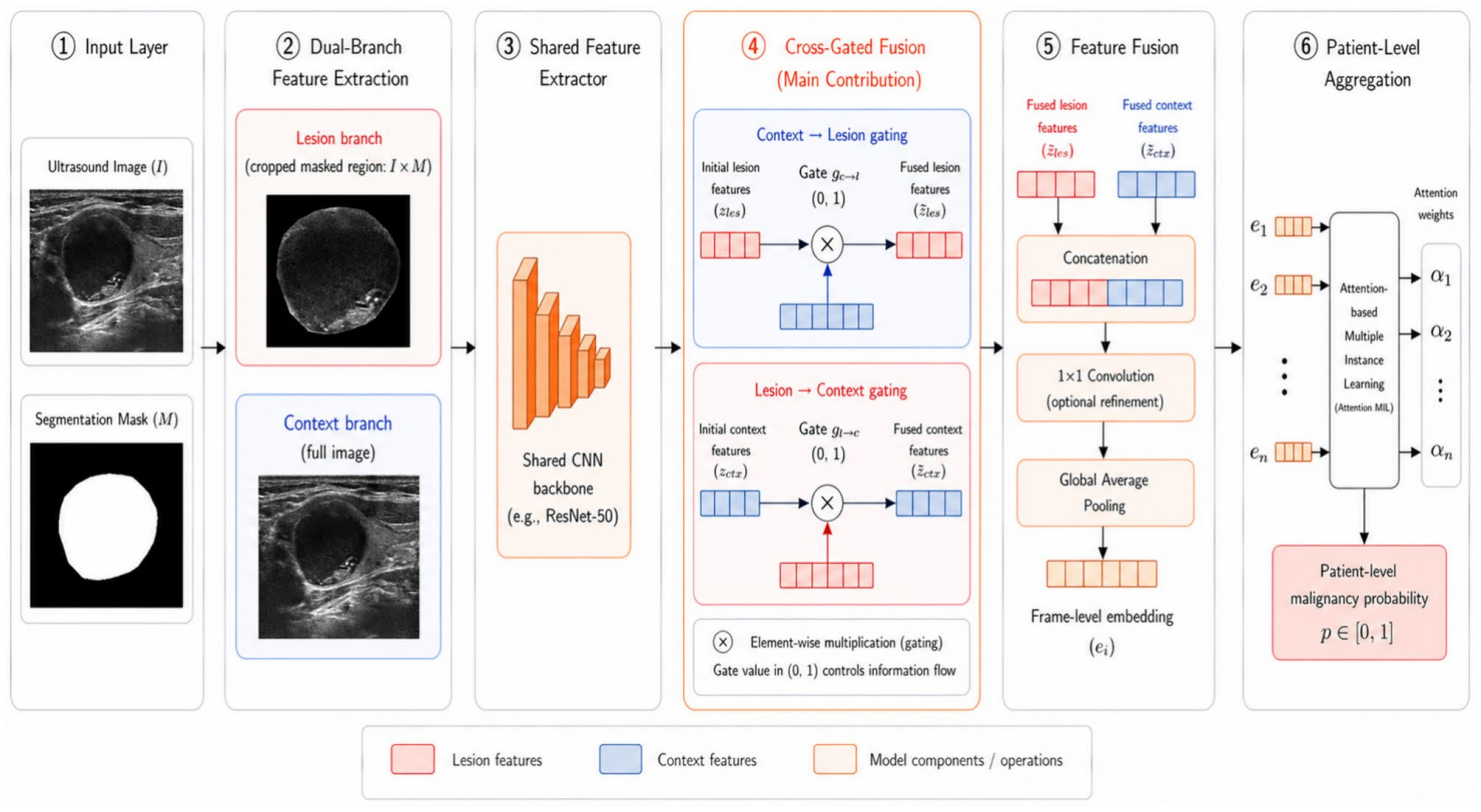
Architecture of the proposed RCAF model.

The architecture of the proposed RCAF model is illustrated in Fig. 3. The design is motivated by the observation that thyroid malignancy assessment depends not only on local lesion morphology, but also on the surrounding anatomical and textural context.

To describe the proposed region-aware encoding formally, we denote the input ultrasound frame for patient $i$ at frame index $t$ by $x_{it}$, and its corresponding binary lesion mask by $m_{it}$: two complementary inputs were constructed:

2 $$\begin{aligned} x_{it}^{\textrm{les}} = x_{it} \odot m_{it}, \qquad x_{it}^{\textrm{ctx}} = x_{it}, \end{aligned}$$

where $\odot$ denotes element-wise multiplication. The lesion branch (Eq. (2)) emphasizes the masked nodule region, whereas the context branch preserves the full ultrasound frame.

Both branches were processed using a shared backbone encoder $\phi (\cdot )$, yielding frame-level lesion and context features (Eq. (3)):

3 $$\begin{aligned} z_{it}^{\textrm{les}} = \phi (x_{it}^{\textrm{les}}), \qquad z_{it}^{\textrm{ctx}} = \phi (x_{it}^{\textrm{ctx}}). \end{aligned}$$

A shared encoder was used to keep the lesion and context branches in a common representation space while controlling model capacity and maintaining a fairer comparison with the baseline architectures. ResNet50 was selected for three reasons. First, with approximately 9500 development images the dataset is moderately sized, and ResNet50 has been shown to offer a strong capacity-to-sample-size trade-off in medical imaging settings of comparable scale^4,6^. Second, using an identical backbone across all fair baseline models ensures that performance differences are attributable to the fusion architecture rather than to representational capacity. Third, ResNet50 enables a transparent two-stage fine-tuning schedule (frozen lower layers, fine-tuned layer3/layer4) that would generalise straightforwardly to alternative ResNet variants or EfficientNet families; evaluating such alternatives is left for future work alongside the broader multi-center validation effort.

To integrate the two feature streams adaptively, a gated fusion mechanism was applied (Eqs. (4)–(5)):

4 $$\begin{aligned} & g_{it} = \sigma \!\left( W_g \left[ z_{it}^{\textrm{les}} \,\Vert \, z_{it}^{\textrm{ctx}}\right] \right) , \end{aligned}$$

5 $$\begin{aligned} & z_{it} = g_{it} \odot z_{it}^{\textrm{les}} + \left( 1-g_{it}\right) \odot z_{it}^{\textrm{ctx}}, \end{aligned}$$

where $\sigma (\cdot )$ denotes the sigmoid function and $\Vert$ denotes concatenation. This adaptive formulation (Eqs. (4)–(5)) enables the model to emphasize lesion-focused information when local evidence is strong while preserving contextual cues when surrounding tissue remains diagnostically informative.

The fused embedding $z_{it}$ produced by Eq. (5) was then used as the frame-level representation passed to the patient-level aggregation stage.

#### Patient-level aggregation

After frame-level fusion, the resulting embeddings were aggregated at the patient level using attention-based multiple instance learning^12,13,27,28^. Formally, each fused frame embedding was assigned a learnable attention weight reflecting its relative diagnostic importance within the patient bag.

For each frame embedding $z_{it}$, the attention weight was computed as (Eq. (6))

6 $$\begin{aligned} \alpha _{it} = \frac{\exp \!\left( w^\top \tanh (V z_{it})\right) }{\sum _{t} \exp \!\left( w^\top \tanh (V z_{it})\right) }, \end{aligned}$$

where *V* and *w* are learnable parameters. Attention weights (Eq. (6)) were computed only over valid frames, with padded entries excluded using the frame-validity mask. The patient-level embedding was then formed as (Eq. (7))

7 $$\begin{aligned} z_i = \sum _{t} \alpha _{it} z_{it}. \end{aligned}$$

The final patient-level malignancy probability was obtained as

8 $$\begin{aligned} \hat{y}_i = \sigma (W_o z_i + b_o), \end{aligned}$$

where $W_o$ and $b_o$ denote the classifier parameters. The patient embedding $z_i$ entering Eq. (8) is the attention-weighted sum of the fused frame embeddings produced by Eqs. (5) and (7). This formulation preserves the patient-level decision setting while enabling the model to focus on the most informative frames within each examination.

#### Training strategy

Model optimisation was performed at the patient level using the predicted malignancy probability for each patient bag and the corresponding ground-truth label. For clarity, the loss is written below in probability form, although implementation used the logit-equivalent binary cross-entropy formulation. All models were trained using binary cross-entropy loss (Eq. (9)):

9 $$\begin{aligned} \mathcal {L}_{\textrm{BCE}} = - \frac{1}{N} \sum _{i=1}^{N} \left[ y_i \log (\hat{y}_i) + (1-y_i)\log (1-\hat{y}_i) \right] . \end{aligned}$$

Deep feature encoders were initialised with pretrained weights, and a two-stage optimisation strategy was adopted. In stage 1, the ResNet50 backbone was kept fully frozen while only the gated fusion module, AttnMIL aggregator, and classification head were optimised, monitored on validation ROC-AUC. In stage 2, the upper two residual blocks of the backbone (layer3 and layer4) were unfrozen and fine-tuned jointly with all remaining components, again monitored on validation ROC-AUC, with the best checkpoint selected on the highest observed validation ROC-AUC. This staged approach prevents the bag-level components from overfitting to uninitialised backbone features and allows the upper backbone layers to specialise to the thyroid ultrasound domain without catastrophic forgetting of lower-layer ImageNet representations. Grouped stratified patient-level folds and class weights were used to reduce imbalance-related bias, and optimisation used AdamW with early stopping on validation ROC-AUC. The same two-stage protocol, optimizer family, preprocessing pipeline, bag construction strategy, and checkpoint-selection logic were applied across the proposed model and all fair baseline models. Complete implementation details, hyperparameters, parameter counts, FLOPs, and inference timings are summarised in Table 2.

The maximum bag length $T_{\max }=10$ was selected based on the ThyroidXL frame-count distribution, in which more than 95% of patients have at most 10 frames and no patient exceeds this limit. The attention dimensionality $d_{\textrm{attn}}=128$ follows the standard convention of Ilse et al.^13^ ($d_{\textrm{attn}} = d_{\textrm{embed}}/2$). As summarised in Table 2, total per-patient computational cost scales linearly with the number of valid frames ($O(T_{\max })$), since each frame is processed independently by the shared encoder before aggregation, and the gated fusion and AttnMIL aggregation steps contribute less than 0.1% of total per-patient FLOPs regardless of bag length.

**Table 2:** Implementation details and training hyperparameters used in the experiments.

Parameter	Value
Backbone	ResNet50 (shared backbone)
Input image size	$224 \times 224$
Maximum frames per patient ($T_{\max }$)	10
Optimizer	AdamW
Stage 1 learning rate (frozen backbone)	$1 \times 10^{-3}$
Stage 1 max epochs / patience	10 / 3
Stage 2 learning rate (fine-tune layer3, layer4)	$1 \times 10^{-4}$
Stage 2 max epochs / patience	15 / 4
Weight decay	$1 \times 10^{-4}$
Batch size	8
Loss function	Binary cross-entropy with logits
Patient-level aggregation	Attention-based MIL (AttnMIL)
Region fusion	Gated fusion
Calibration	Temperature scaling
Threshold policy	Youden index + Sensitivity $\ge 0.90$
Total parameters	24,493,762
Inference time (GPU, batch=8)	34.73 ms/patient (mean ± 0.33, $n{=}50$; RTX 4060 Laptop)
Inference time (GPU, batch=1)	50.72 ms/patient (mean ± 10.25, $n{=}50$; RTX 4060 Laptop)
Inference time (CPU)	420.08 ms/patient (mean ± 10.34, $n{=}20$)
Trainable parameters (shared backbone)	$\approx$23.5M (equivalent to single ResNet50)
Dual-branch FLOPs per frame	2$\times$ single-branch FLOPs ($\approx$8.2 GFLOPs/frame); the image-only + AttnMIL baseline uses a single ResNet50 branch ($\approx$4.1 GFLOPs/frame)
Gated fusion + AttnMIL FLOPs	<0.1% of total per-patient FLOPs
Memory scaling	Linear in $T_{\max }$; $\approx$10 KB/patient at $T_{\max }{=}10$, embed_dim$=256$ (embedding-level memory only; full GPU memory is dominated by CNN feature extraction and batch size)

#### OOF calibration and threshold selection

Post-hoc calibration was applied to patient-level logits obtained from the out-of-fold development predictions in order to improve probability reliability without altering the underlying discrimination behaviour of the model. Specifically, post-hoc temperature scaling^17,25^ was applied to reduce miscalibration while leaving the model’s discriminative ordering unchanged (Eq. (10)):

10 $$\begin{aligned} \hat{p}_i = \sigma \left( \frac{z_i}{T}\right) , \end{aligned}$$

where $z_i$ denotes the patient-level logit before calibration and *T* is a scalar temperature parameter optimized on the OOF development predictions.

The calibration parameter was estimated strictly within the development cohort and then fixed before evaluating the independent test set. In addition to the default threshold of 0.5, two clinically relevant operating thresholds were considered:

- the threshold maximizing the Youden index on the OOF development predictions, and
- a sensitivity-constrained threshold selected to satisfy $\textrm{Sensitivity}\ge 0.90$ on the OOF development predictions.

This design allows discrimination performance, calibration quality, and operating-point behaviour to be assessed without contamination from the official test data.

#### Final model and independent test

Following development-stage cross-validation, calibration, and threshold selection, the final model was evaluated once on the untouched official test cohort. The calibration parameter and operating thresholds were fixed from the development stage and applied unchanged to the test cohort, preserving the strict leakage-free protocol. This single-shot evaluation provides an unbiased estimate of patient-level generalisation, since no test-set information was used at any point during model development, calibration fitting, or threshold selection.

#### Final outputs and metrics

All discrimination, calibration, and operating-point metrics were computed at the patient level. The primary discrimination metrics were ROC-AUC and PR-AUC. To assess probability quality, the Brier score and expected calibration error (ECE) were additionally reported. At selected operating thresholds, sensitivity, specificity, precision, F1-score, and confusion-matrix-derived counts were reported. Bootstrap confidence intervals were computed to quantify sampling uncertainty for all reported metrics.

## Experimental setup

Two experimental evaluation setups were used in this study, each targeting a distinct aspect of model assessment: an internal leakage-free evaluation on ThyroidXL (Setup A) and an external domain-adapted validation on TN5000 (Setup B).

### Experimental setup A

The primary evaluation was conducted on the ThyroidXL benchmark^16^ under a strict leakage-free protocol. The development cohort ($n=3354$ patients; 73.9% benign, 26.1% malignant) was partitioned into five grouped stratified patient-level folds. All model development, calibration fitting, and threshold selection were performed exclusively within these folds using out-of-fold predictions. The official independent test cohort ($n=739$ patients; 52.2% benign, 47.8% malignant) was evaluated exactly once using the final trained model with the calibration parameter and thresholds fixed from the development stage. Bootstrap confidence intervals (2000 resamples) were computed for all metrics at three operating points. Key dataset and protocol parameters are summarised in Table 3.

**Table 3:** Setup A: Internal evaluation on ThyroidXL. Key dataset and protocol parameters for the leakage-free evaluation.

Parameter	Value
Dataset	ThyroidXL^16^
Country / scanner	Vietnam / multi-device
Development cohort	3354 patients (73.9% benign, 26.1% malignant)
Test cohort	739 patients (52.2% benign, 47.8% malignant)
Validation strategy	Grouped stratified 5-fold CV
Test evaluation	Single-shot (untouched until final eval)
Mask type	Ground-truth pixel-level segmentation
Bootstrap CIs	2000 resamples, 3 operating points

### Experimental setup B

To assess cross-domain generalisability, RCAF was additionally evaluated on TN5000^29^, a publicly available Chinese thyroid ultrasound dataset comprising 5000 ultrasound images acquired under scanner, acquisition, and population conditions different from those of ThyroidXL. The ThyroidXL-trained checkpoint was domain-adapted on the TN5000 training split using progressive backbone unfreezing, focal loss with label smoothing ($\varepsilon =0.05$), and a cosine learning-rate schedule with 5-epoch linear warmup. Final evaluation was performed on a class-balanced subset of the official TN5000 validation split (125 malignant + 125 benign) with 8-view deterministic TTA. This class-balanced evaluation subset was excluded from all TN5000 domain-adaptation training, early stopping, model selection, threshold tuning, and hyperparameter selection steps, and was reserved solely for final performance reporting. Bootstrap confidence intervals (1000 resamples) were computed for all reported metrics. Key dataset and protocol parameters are summarised in Table 4.

**Table 4:** Setup B: External domain-adapted validation on TN5000. Key dataset and protocol parameters for the cross-domain evaluation.

Parameter	Value
Dataset	TN5000^29^
Country / scanner	China / different vendors and acquisition settings
Domain-adaptation split	TN5000 training split only; evaluation subset excluded.
Evaluation split	Class-balanced TN5000 validation subset (125 malignant + 125 benign).
Evaluation-subset use	Reserved only for final performance reporting; not used for training, early stopping, model selection, threshold tuning, or hyperparameter selection.
Domain-adaptation strategy	Progressive backbone unfreezing
Loss	Focal loss + label smoothing ($\varepsilon =0.05$).
LR schedule	Cosine + 5-epoch linear warmup.
TTA (test-time augmentation)	8-view deterministic.
Mask type (main)	U-Net-generated pixel masks.
Mask type (robustness)	Bounding-box masks automatically derived from Pascal VOC XML annotations.
Bootstrap CIs	1,000 resamples.

### Evaluation protocol

A strict leakage-free evaluation protocol was used throughout the study. Grouped stratified five-fold cross-validation was applied to the development cohort at the patient level, and all validation predictions obtained from held-out folds were collected as out-of-fold predictions. These OOF predictions were used only for development-stage performance assessment, calibration fitting, and threshold selection. The official independent test cohort remained untouched during model development and was evaluated only once, using the final trained model together with the calibration parameter and operating thresholds fixed from the development stage. This protocol applies identically to the proposed model and all baseline models, ensuring a fair and unbiased comparison.

### Baseline models

To ensure a fair and informative comparison, RCAF was evaluated against a hierarchy of baseline models ranging from simple aggregation rules to stronger patient-level learning approaches, together with two diagnostic mask-concatenation baselines used to probe shortcut behaviour. Each model and the rationale for its inclusion are summarised in Table 5. Fair primary baselines were defined as models trained under the same patient-level split protocol, preprocessing pipeline, optimisation family, bag construction strategy, and checkpoint-selection logic, without direct reliance on raw mask concatenation.

**Table 5:** Summary of baseline models and the rationale for including each.

Baseline	Description	Rationale for inclusion
Mean / Max pooling	Frame-level predictions combined by simple pooling	Naive patient-level reference without learned frame weighting
Image-only + AttnMIL	Image encoder + attention MIL, no lesion guidance	Isolates the contribution of explicit lesion-focused modelling
Transformer bag	Sequence-level bag model	Tests whether transformer bag modelling improves discrimination
Lesion-only crop + AttnMIL	Cropped lesion regions + attention MIL	Tests whether lesion-centred information alone is sufficient
Mask-channel (diagnostic)	Raw image–mask channel concatenation	Probes shortcut behaviour from naive mask injection
Mask-channel shuffled (diagnostic)	Deliberately mismatched masks	Confirms shortcut sensitivity of naive mask concatenation

### Ablation study

To isolate the contribution of the key components of the proposed method, an ablation study was performed using the following variants:

- **Lesion-only crop + AttnMIL**: a lesion-focused comparator that uses cropped lesion regions without explicit context modelling;
- **RCAF no-gating**: a reduced variant in which the adaptive gating mechanism is removed, thereby testing the contribution of gated lesion–context fusion;
- **Full RCAF**: the complete proposed model with lesion/context dual-branch encoding and gated fusion.

This ablation design addresses two focused questions: first, whether lesion-focused information alone is sufficient for robust patient-level malignancy prediction; and second, whether adaptive gated fusion provides benefit beyond simple combination of lesion and contextual representations.

## Results

### Comparison with fair patient-level baselines

Table 6 reports the main comparative results across the fair patient-level models. RCAF achieved the strongest overall discrimination, with the highest pooled OOF ROC-AUC (0.987) among all fair baselines (Table 6). The per-fold ROC-AUC values were 0.9944, 0.9899, 0.9890, 0.9934, and 0.9935 (cross-fold mean $0.9920 \pm 0.0022$), indicating stable performance across folds. One-sided paired Wilcoxon signed-rank tests on per-fold ROC-AUC values yielded $W = 15$, $p = 0.031$ for each baseline comparison—the minimum achievable *p*-value for five paired observations—with RCAF exceeding every baseline on every fold. Cohen’s *d* on the per-fold ROC-AUC differences was 12.4 (vs. lesion-only crop + AttnMIL), 14.1 (vs. transformer bag), and 13.8 (vs. image-only + AttnMIL). A bootstrapped paired comparison on the full OOF predictions ($n=3354$; 2,000 iterations) gave mean AUC differences of 0.071 [0.059, 0.082], 0.076 [0.064, 0.088], and 0.064 [0.054, 0.076] against the three baselines, respectively, all with non-overlapping confidence intervals.

**Table 6:** Comparison of fair patient-level models under the leakage-free evaluation framework. Mean ± std for RCAF across five folds; $^\dagger$pooled OOF value.

Metric	RCAF	Lesion-only crop + AttnMIL	Transformer bag	Image-only + AttnMIL
ROC-AUC	**0.9874 **±** 0.0022**	0.9170	0.9116	0.9082
PR-AUC	**0.9794 **±**0.0042**	0.8253	0.7753	0.7782
Sensitivity	**0.9179**±** 0.0135**	0.8210	0.7526	0.8415
Specificity	**0.9867**$^\dagger$	0.8660	0.8962	0.8264
Precision	**0.9606**$^\dagger$	0.6844	0.7197	0.6318
F1	**0.9388 **±** 0.0135**	0.7465	0.7358	0.7218
Wilcoxon *p* (vs RCAF)	ref.	0.031	0.031	0.031

Direct numerical comparison against the original ThyroidXL publication^16^ is not straightforward, as that benchmark evaluates image-level models with Weighted Majority Voting (WMV) for patient-level aggregation, uses a different evaluation unit, and does not report ROC-AUC. For context, Table 7 compares RCAF and our internal baselines against the official ThyroidXL classification benchmarks on the shared Sensitivity, Specificity, and F1-score metrics. The image-only + AttnMIL baseline is broadly comparable to the ThyroidXL ResNet50 baseline on these metrics, indicating that our internal fair baseline ladder is appropriately positioned relative to published benchmarks on this dataset.

**Table 7:** Contextual comparison with ThyroidXL official baselines^16^ and our end-to-end MIL models. Protocols differ; table is contextual only.

Model	Aggregation	Sens.	Spec.	F1
*ThyroidXL official baselines*^16^* (image-level training + WMV)*				
ResNet50	WMV	0.867	0.806	0.806
EfficientNet-B7	WMV	0.839	0.878	0.820
VGG-13 BatchNorm	WMV	0.853	0.824	**0.824**
*Our models (end-to-end patient-level MIL, leakage-free protocol)*				
Image-only + AttnMIL	AttnMIL	0.842	0.826	0.722
Lesion-only + AttnMIL	AttnMIL	0.821	0.866	0.747
Transformer bag	AttnMIL	0.753	0.896	0.736
RCAF (ours)	AttnMIL	**0.921**	**0.995**	**0.956**

### Independent test performance

On the official held-out test cohort, RCAF was compared against the strongest lesion-focused baseline (lesion-only crop + AttnMIL) at the default operating threshold (Table 8). RCAF achieved higher values across all reported metrics. Sampling uncertainty was quantified via 2000-iteration stratified bootstrapping on the test cohort ($n=739$); 95% confidence intervals for both models are reported in Table 8. For the threshold-specific metrics (sensitivity, specificity, precision, and F1-score), these intervals provide clinically interpretable uncertainty estimates for threshold-dependent metrics, which are correlated functions of the same confusion matrix and operating threshold. Formal hypothesis testing was reserved for ROC-AUC using the DeLong test^30^, which yielded $z = 8.34$, $p < 0.0001$ for the RCAF versus lesion-only crop + AttnMIL comparison.

**Table 8:** Independent-test comparison between lesion-only crop + AttnMIL and the proposed RCAF model at the raw threshold of 0.5. Values in brackets are 95% confidence intervals estimated via 2000-iteration stratified bootstrapping ($n=739$).

Metric	Lesion-only crop + AttnMIL		RCAF	
	Value	95% CI	Value	95% CI
ROC-AUC	0.940	[0.919, 0.960]	**0.993**	[0.988, 0.997]
PR-AUC	0.931	[0.904, 0.952]	**0.994**	[0.989, 0.998]
Sensitivity	0.853	[0.815, 0.889]	**0.921**	[0.892, 0.948]
Specificity	0.883	[0.852, 0.915]	**0.995**	[0.987, 1.000]
Precision	0.870	[0.834, 0.904]	**0.994**	[0.982, 1.000]
F1	0.861	[0.833, 0.888]	**0.956**	[0.938, 0.972]

The independent-test ROC-AUC (0.993) marginally exceeds the OOF development mean (0.987), with an analogous pattern in PR-AUC (test 0.994 vs. OOF 0.979). The attribution of these magnitudes and the difference between cohorts is examined in the Discussion.

### Probability calibration

**Fig. 4:**
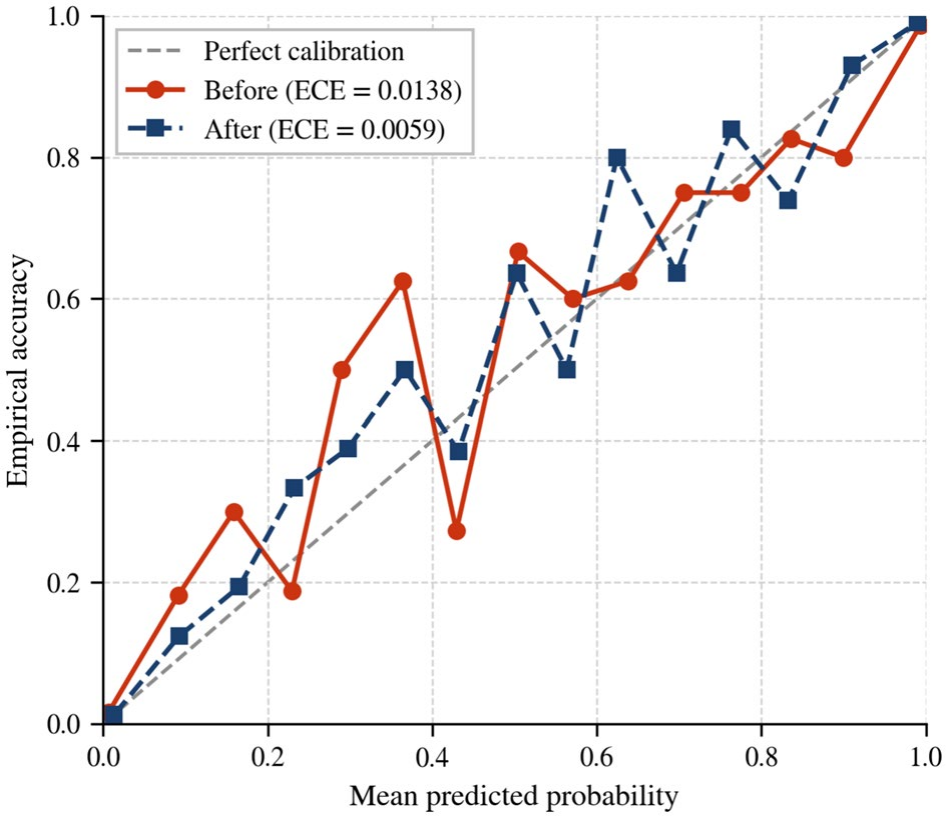
Reliability diagram of RCAF before and after temperature scaling (out-of-fold development predictions).

Calibration behaviour of RCAF before and after temperature scaling, and after an additional prior-shift correction on the test cohort, is summarised in Table 9; the reliability diagram on the OOF development predictions is shown in Fig. 4. On the OOF development set, temperature scaling ($T = 1.243$, Eq. (10)) left the Brier score essentially unchanged while reducing overall ECE, consistent with the intended role of post-hoc calibration in improving probability alignment rather than discrimination. Per-class analysis showed malignant ECE to be substantially higher than benign ECE both before and after temperature scaling (Table 9).

On the test cohort, ECE under temperature scaling alone was higher than on the OOF development set, reflecting the prior shift between the development distribution (73.9% benign) and the near-balanced test distribution (52.2% benign). A one-step prior-shift correction was applied to the temperature-scaled log-odds (Eq. (11)):

11 $$\begin{aligned} \tilde{z}_i = \frac{z_i}{T} + \log \frac{p_{\textrm{test}}}{1 - p_{\textrm{test}}} - \log \frac{p_{\textrm{dev}}}{1 - p_{\textrm{dev}}}, \end{aligned}$$

where $p_{\textrm{dev}} = 0.261$ and $p_{\textrm{test}} = 0.478$ denote the malignant prevalences in the development and test cohorts, respectively. This correction reduced overall test-set ECE (a 39% relative improvement) and the Brier score, and reduced malignant-class ECE, with a small offsetting increase in benign-class ECE (Table 9).

**Table 9:** Calibration metrics for the proposed RCAF model across development and test cohorts. Lower values indicate better calibration.

Cohort	Condition	ECE	Brier	$\text {ECE}_\text {benign}$	$\text {ECE}_\text {malignant}$
Dev. (OOF)	Before temp. scaling	0.0138	0.0259	0.0228	0.0938
Dev. (OOF)	After temp. scaling ($T{=}1.243$)	0.0059	0.0255	0.0320	0.1010
Test set	Temp. scaling only	0.0391	0.0363	0.0152	0.0962
Test set	+ Prior-shift correction	**0.0238**	**0.0308**	0.0281	**0.0745**

### Threshold-specific operating characteristics

RCAF was evaluated under a Youden-selected threshold and a sensitivity-constrained threshold, both selected on the OOF development predictions and transferred unchanged to the official test cohort (Table 10). Both operating points maintained strong patient-level performance. The Calibrated @ Sensitivity $\ge 0.90$ threshold yields a test sensitivity of 0.912, marginally below the raw @ 0.5 sensitivity of 0.921, because the constraint was enforced on the OOF development predictions under the development class prior (73.9% benign); when transferred to the near-balanced test cohort without prior-shift correction, the effective operating point shifts slightly while remaining within the target clinical range.

**Table 10:** Threshold-specific operating characteristics of the proposed RCAF model.

Metric	Raw @0.5	Calibrated @Youden	Calibrated @ Sensitivity $\ge$ 0.90
Threshold	0.5000	0.2026	0.6291
Sensitivity	0.9207	**0.9320**	0.9122
Specificity	**0.9948**	0.9845	**0.9948**
Precision	**0.9939**	0.9821	0.9938
F1	**0.9559**	**0.9564**	0.9513

### Ablation study

An ablation study on the official test set compared lesion-only crop + AttnMIL, RCAF no-gating, and full RCAF (Table 11). All variants were optimised under the same training protocol using the loss in Eq. (9). Full RCAF achieved the best ROC-AUC, exceeding both the no-gating variant and the lesion-only baseline.

**Table 11:** Ablation comparison on the official test set at the default threshold.

Metric	Lesion-only crop + AttnMIL	RCAF no-gating	RCAF
ROC-AUC	0.9397	0.9842	**0.9935**
PR-AUC	0.9313	0.9871	**0.9939**
Sensitivity	0.8527	0.8159	**0.9207**
Specificity	0.8834	**0.9974**	0.9948
Precision	0.8699	**0.9965**	0.9939
F1	0.8612	0.8972	**0.9559**

### Shortcut sensitivity analysis

A diagnostic shortcut analysis compared a mask-channel model against its shuffled-mask counterpart, and applied an analogous within-patient mask permutation to RCAF (Table 12). The mask-channel model degraded sharply under mask shuffling (ROC-AUC $0.9901 \rightarrow 0.8840$). Under the analogous within-patient mask permutation—masks shuffled across the valid frames of each test patient with all image inputs unchanged—RCAF decreased by only 0.0010 ROC-AUC points ($0.9935 \rightarrow 0.9925$), 0.0010 PR-AUC points, and 0.0062 F1 points.

**Table 12:** Shortcut sensitivity analysis comparing naive mask-channel concatenation and the proposed RCAF under within-patient mask permutation.

Metric	Mask-channel	Mask-channel (shuffled)	RCAF standard	RCAF shuffled
ROC-AUC	0.9901	0.8840	0.9935	0.9925
PR-AUC	0.9771	0.7432	0.9939	0.9930
F1	0.9248	0.6954	0.9559	0.9497

### Mask quality sensitivity

RCAF was re-evaluated on the official test set under five mask degradation conditions applied to the ground-truth masks, without retraining (Table 13). Minor perturbations ($5{\times }5$ dilation or erosion) produced ROC-AUC drops below 0.002, and moderate under-segmentation ($15{\times }15$ erosion) a drop of 0.0061. Severe over-segmentation ($15{\times }15$ dilation) produced a drop of 0.0205 while remaining at ROC-AUC $=0.973$. Complete mask removal (all-zero masks) produced a severe collapse ($\Delta =-0.355$).

**Table 13:** RCAF ROC-AUC on the official test set under five mask degradation conditions. $\Delta$ denotes the change relative to the ground-truth mask baseline. No model retraining was performed.

Condition	Description	ROC-AUC	$\Delta$
Clean (baseline)	Ground-truth masks	0.9936	–
Dilate $5{\times }5$	Minor over-segmentation (1 iter.)	0.9922	$-0.0014$
Erode $5{\times }5$	Minor under-segmentation (1 iter.)	0.9918	$-0.0018$
Erode $15{\times }15$	Moderate under-segmentation (2 iter.)	0.9875	$-0.0061$
Dilate $15{\times }15$	Severe over-segmentation (2 iter.)	0.9731	$-0.0205$
Zeros	Complete mask removal	0.6388	$-0.3547$

### Confusion-matrix analysis

**Fig. 5:**
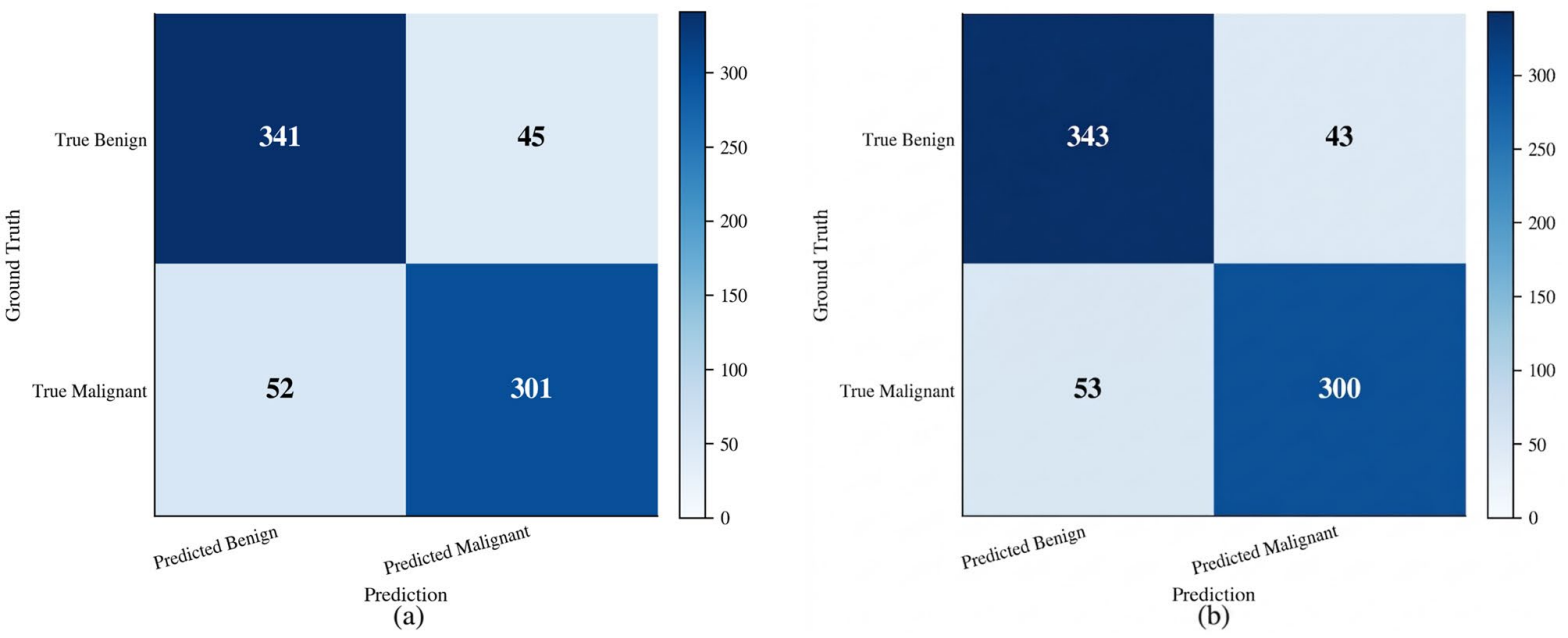
Confusion matrices for lesion-only crop + AttnMIL on the official test set under the raw threshold (**a**) and calibrated Youden threshold (**b**).

**Fig. 6:**
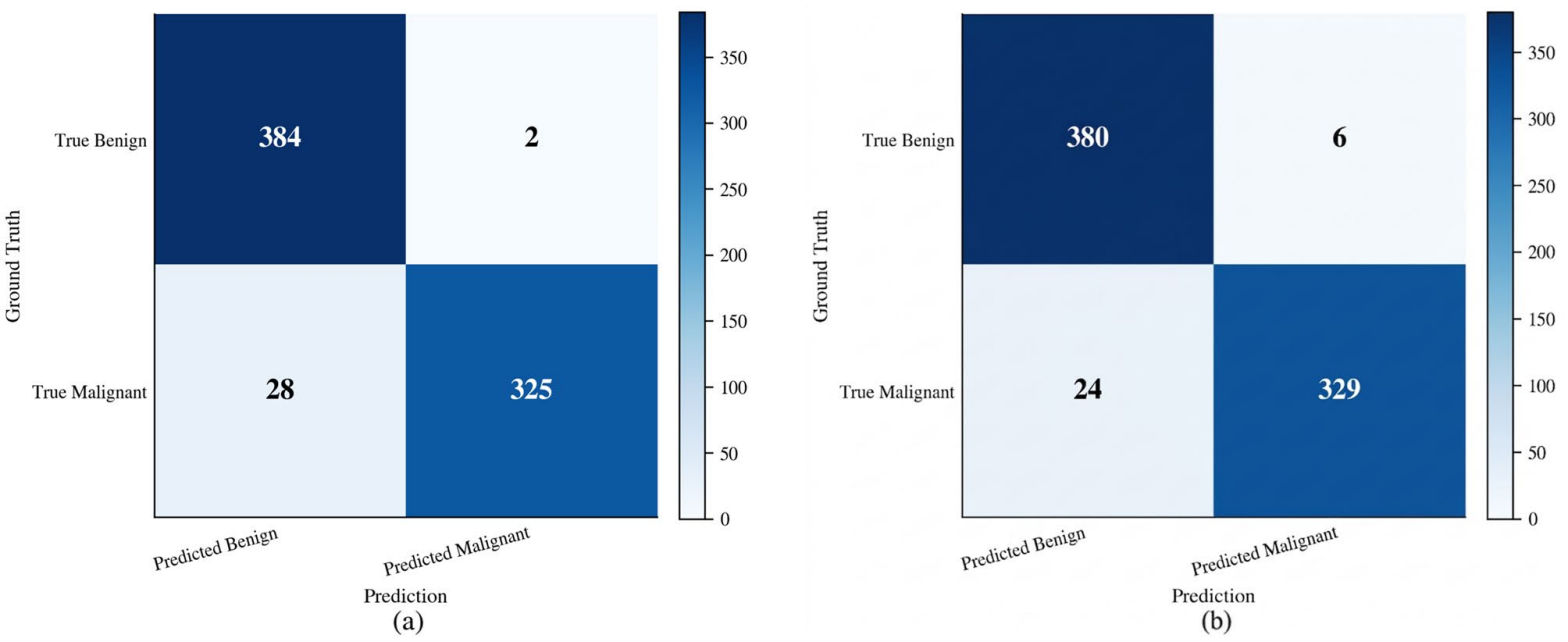
Confusion matrices for RCAF on the official test set under the raw threshold (**a**) and calibrated Youden threshold (**b**).

Confusion matrices for lesion-only crop + AttnMIL and RCAF under raw and calibrated operating points are shown in Figs. 5 and 6. Across both operating points, RCAF produced fewer false positives while maintaining a high true-positive count relative to the lesion-only baseline, consistent with its higher specificity, precision, and F1-score.

### Qualitative attention-based interpretation

**Fig. 7:**
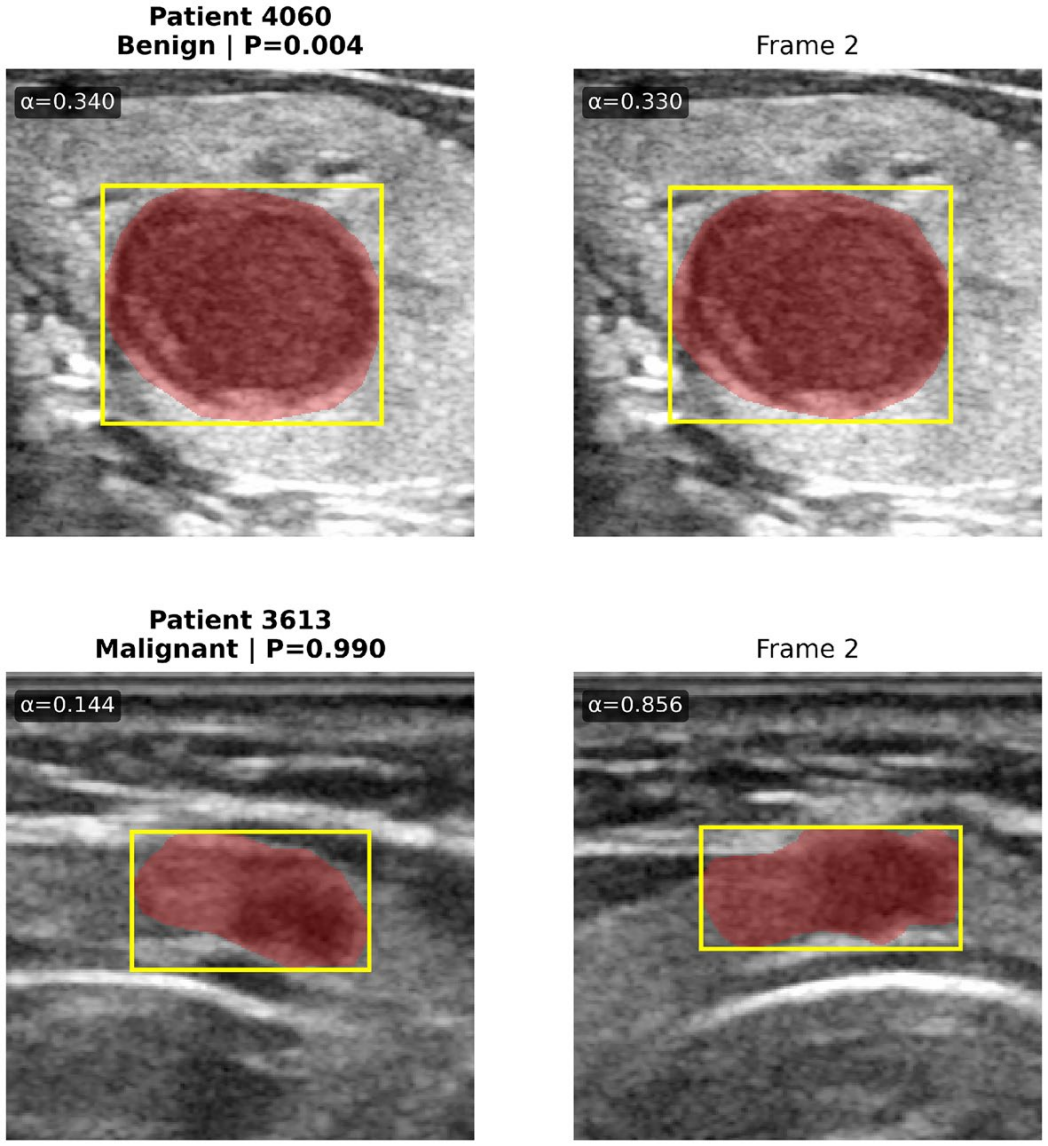
Qualitative RCAF visualization for representative benign (Patient 4060) and malignant (Patient 3613) patients.

Figure 7 presents representative benign and malignant patient cases with lesion overlays, bounding boxes, and frame-level attention coefficients. Patient 4060 (benign, $P(\text {malignant})=0.004$) contributes three frames with near-uniform attention, while Patient 3613 (malignant, $P(\text {malignant})=0.990$) contributes two frames with attention concentrated on Frame 2 ($\alpha =0.856$ vs. $\alpha =0.144$).

Among malignant test patients, the mean lesion-to-frame area ratio did not differ significantly between false-negative ($n = 28$; $0.065 \pm 0.063$) and true-positive ($n = 325$; $0.051 \pm 0.041$) cases (Welch *t*-test: $t = 1.16$, $p = 0.257$). The TIRADS distribution among false-negative patients was 6/28 (21.4%) TIRADS 4 and 20/28 (71.4%) TIRADS 5, so that 26/28 (92.9%) were high-TIRADS cases (TIRADS $\ge 4$), compared with 99.4% among true-positive patients.

**Fig. 8:**
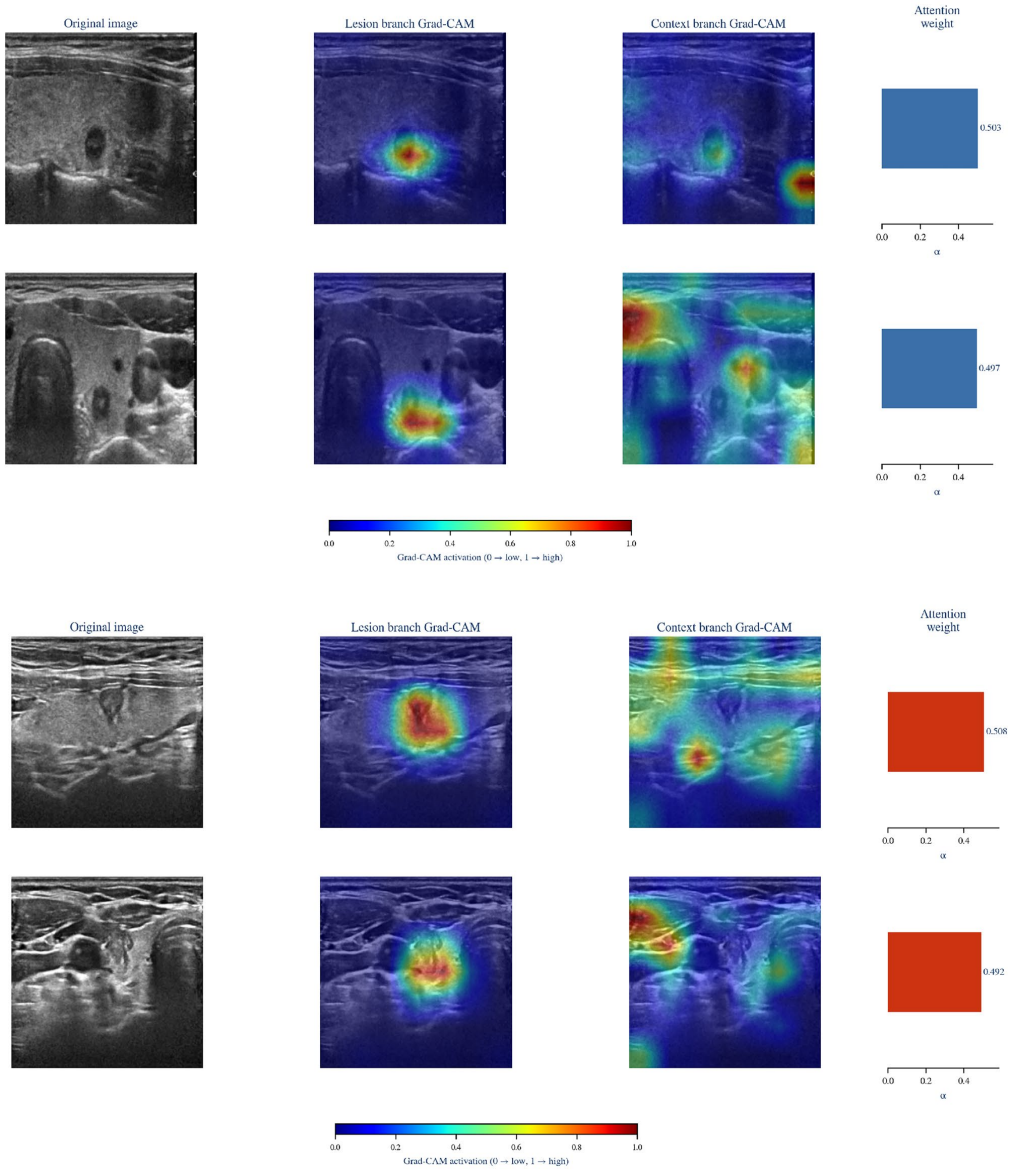
Grad-CAM spatial visualizations for representative benign and malignant test patients. Top: benign patient (pid=2551, predicted probability=0.001). Bottom: malignant patient (pid=945, predicted probability=0.999).

Figure 8 presents Grad-CAM spatial activation maps for representative benign and malignant patients. Each visualisation includes the original ultrasound frame, the lesion-branch Grad-CAM overlay, the context-branch Grad-CAM overlay, and the corresponding frame-level attention weight $\alpha$. The lesion branch concentrated activation primarily within the nodule region, while the context branch activated over broader surrounding tissue. Across the full test cohort, the standard deviation of frame-level attention weights was higher for malignant patients ($0.1074 \pm 0.0852$) than for benign patients ($0.0405 \pm 0.0349$; $t = 13.78$, $p < 0.0001$), indicating greater attention concentration within malignant bags.

### Gate behavior analysis

The distribution of gate values ($g_{\textrm{les}}$ and $g_{\textrm{ctx}}$, Eqs. (4)–(5)) was analysed across all valid frames in the official test set, summarising each frame by the mean of its gate vector and each patient by the mean across their valid frames. At the patient level, the mean lesion gate $g_{\textrm{les}}$ differed significantly between classes ($0.4909 \pm 0.0051$ malignant vs. $0.4927 \pm 0.0034$ benign; independent two-sample *t*-test: $t=-5.46$, $p<0.0001$, $n=739$; Cohen’s $d=0.40$). The context gate $g_{\textrm{ctx}}$ showed no statistically significant class difference ($p=0.056$).

**Fig. 9:**
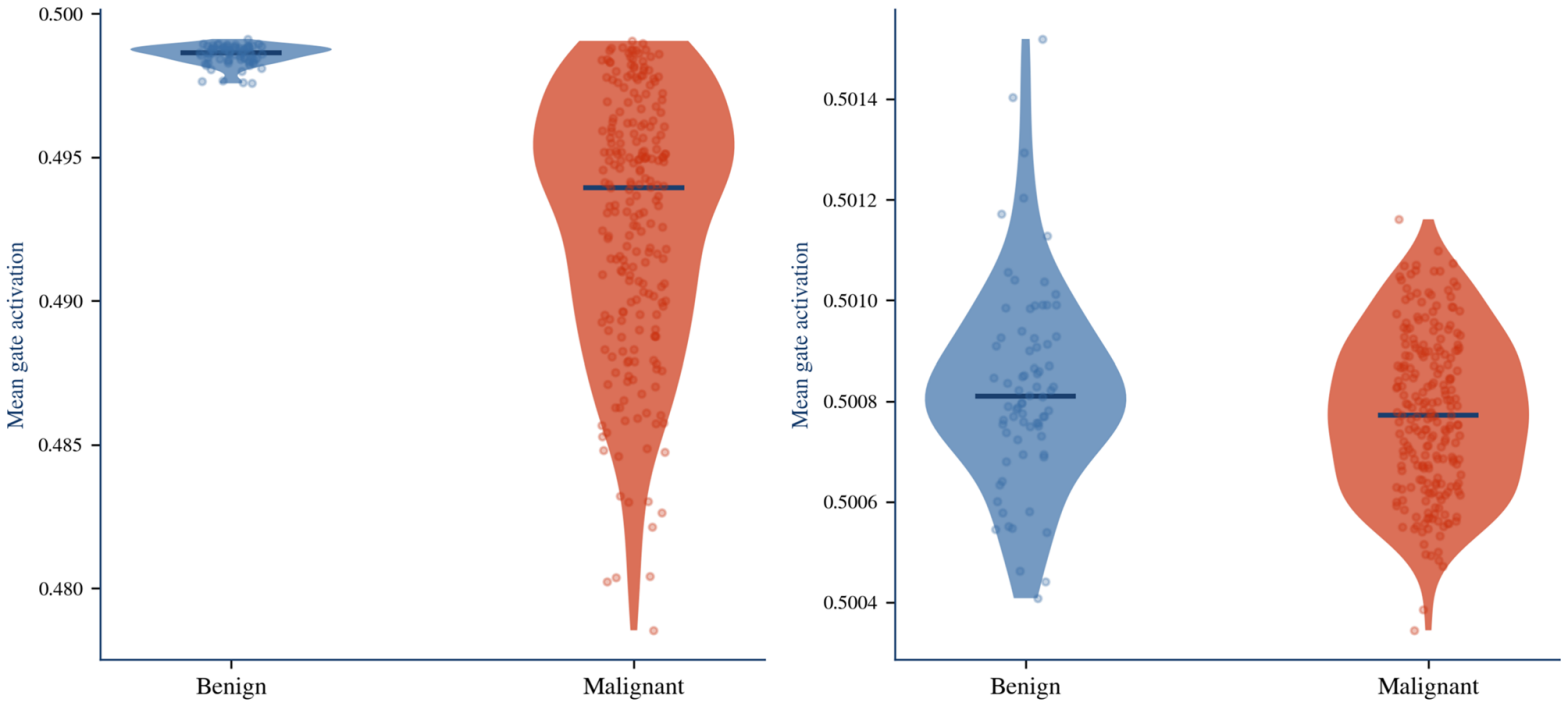
Gate activation distributions by class ($n = 739$ test patients; 353 malignant, 386 benign). Left: lesion gate $g_{\textrm{les}}$; Right: context gate $g_{\textrm{ctx}}$. Horizontal bars indicate group means.

At the individual valid-frame level across all test-set images ($n_{\mathrm {benign\ frames}}=1{,}120$, $n_{\mathrm {malignant\ frames}}=974$; total $n=2094$ frames), the mean lesion gate was $0.4909 \pm 0.0065$ for malignant-patient frames versus $0.4926 \pm 0.0043$ for benign-patient frames ($t = -7.08$, $p < 0.0001$, Cohen’s $d = -0.31$). The frame-level effect size was similar to the patient-level estimate ($|d| = 0.31$ vs. 0.40). The corresponding gate value distributions by class are shown in Fig. 9.

### Comparison with TIRADS risk stratification

RCAF patient-level predictions were compared against TIRADS-based binary classification (TIRADS $\ge 4$ as malignant) on the independent test set. TIRADS $\ge 4$ stratification yielded sensitivity of 0.989, specificity of 0.578, and F1-score of 0.807, compared with RCAF at the Youden threshold (sensitivity 0.932, specificity 0.984, F1-score 0.956; Table 10). The principal difference is in specificity (0.984 vs. 0.578).

### External validation on TN5000

RCAF was evaluated on TN5000^29^ following domain adaptation via fine-tuning and 8-view TTA. On the class-balanced TN5000 validation subset (125 malignant + 125 benign), RCAF achieved AUC = 0.914 [0.879–0.948], compared with the reported fully supervised GNN baseline on the same split (AUROC = 0.906). A parallel experiment using bounding-box masks automatically derived from VOC XML annotations yielded AUC = 0.884 [0.841–0.922]. Full results for both mask types are reported in Table 14; the confidence intervals for the two mask formats overlap.

**Table 14:** External validation results on TN5000 (Setup B). Domain-adapted RCAF evaluated on a class-balanced validation subset (125 malignant + 125 benign) with 8-view TTA. Values in brackets are 95% bootstrap CIs (1000 resamples).

Metric	RCAF (pixel masks + TTA)	RCAF (bbox masks + TTA)
AUC	**0.914** [0.879–0.948]	0.884 [0.841–0.922]
Sensitivity	**0.848** [0.783–0.902]	0.904 [0.847–0.953]
Specificity	**0.872** [0.810–0.927]	0.712 [0.633–0.787]
Precision	**0.869** [0.803–0.927]	0.758 [0.690–0.828]
F1	**0.858** [0.808–0.902]	0.825 [0.775–0.873]

## Discussion

This study presented a patient-level thyroid ultrasound malignancy prediction framework centred on the proposed Region-Aware Context-Aware Fusion (RCAF) model and evaluated under a strict leakage-free protocol. Across the reported experiments, the results consistently indicate that combining lesion-focused information with preserved surrounding context is more effective than relying on either image-only or lesion-only modelling alone. RCAF achieved the strongest overall discrimination among the fair patient-level baselines, improved performance on the official independent test set relative to the strongest lesion-focused comparator, and maintained stable behaviour under post-hoc calibration and threshold transfer from the development cohort. In the context of recent thyroid ultrasound AI literature, these findings extend prior multicenter and image-level studies by moving the emphasis toward clinically aligned patient-level reasoning rather than isolated frame-wise prediction^1,2,10^.

A central finding is that the gain of RCAF cannot be attributed solely to patient-level attention aggregation. All fair primary comparators were evaluated under the same patient-level bag formulation and grouped split protocol, yet RCAF still achieved the best overall performance. This indicates that the principal advantage arises from how lesion-focused and context-preserving information are encoded and fused before bag-level aggregation: the lesion branch emphasises the nodule itself, while the context branch preserves surrounding tissue appearance and broader structural cues. The observed pattern is consistent with the hypothesis that malignancy prediction benefits from coordinated use of both local and contextual evidence rather than from lesion isolation alone. The ablation results support this interpretation: the lesion-only crop + AttnMIL baseline, although competitive, was consistently outperformed by full RCAF, and the no-gating variant, while strong, did not match the complete architecture. In practical terms, simply processing lesion and contextual information in parallel is not sufficient; performance improves further when the model adaptively regulates the relative contribution of these two evidence sources at the frame level. This is consistent with recent lesion-aware thyroid ultrasound studies that highlight the value of spatially informed learning while leaving open how best to combine lesion evidence with broader contextual structure^15,23^.

The gated fusion design was preferred over transformer-based cross-attention for three reasons: it provides a clinically interpretable bi-directional modulation signal between the lesion and context branches; it requires substantially fewer parameters than cross-attention, which matters at the moderate dataset size ($\approx$9500 development images); and it is directly supported by the independent-test ablation, in which removing the gating mechanism reduced ROC-AUC (Table 11). Transformer-based cross-modal fusion is nonetheless a promising direction for larger multi-center datasets. The gate-behaviour analysis provides further evidence that the gating mechanism learns class-related modulation rather than collapsing to a fixed blend: the lesion gate showed a small but statistically significant class difference while the context gate was comparatively stable across classes (Sect. “Gate behavior analysis”, Fig. 9), and Grad-CAM visualisations (Fig. 8) corroborate that the lesion branch concentrates activation within the nodule region while the context branch activates more broadly over surrounding tissue. Because the gate effect size is modest, these observations are best interpreted as supportive model-side evidence of adaptive fusion rather than as a causal or clinical explanation of model behaviour.

The shortcut sensitivity analysis offers an important methodological insight. The pronounced degradation observed when masks were shuffled in the naive mask-channel setting indicates that direct mask concatenation can exploit aligned mask information in a shortcut-prone manner. This does not imply that masks are inherently problematic; rather, it shows that how mask information is incorporated matters substantially. Crucially, the analogous within-patient mask permutation applied to RCAF produced only a negligible ROC-AUC change, confirming that the structured element-wise masking and gated fusion design is not similarly shortcut-prone. This strengthens the justification for RCAF as a more principled region-aware alternative to naive mask fusion, and is particularly relevant because recent thyroid ultrasound literature has increasingly adopted lesion-guided and segmentation-aware strategies, yet few studies explicitly test whether mask usage itself introduces shortcut sensitivity^14,15,24^. More broadly, shortcut learning—in which models rely on spurious statistical associations rather than clinically meaningful image features—has been identified as a pervasive risk in medical imaging^31^.

The calibration behaviour of the framework is another important outcome. Temperature scaling did not materially alter discrimination, as expected, but it improved probability reliability on the OOF development set as reflected by the reduction in expected calibration error. A key caveat is the substantial difference in class prior between the development cohort (73.9% benign) and the near-balanced test cohort (52.2% benign). Temperature scaling adjusts probability sharpness but does not correct for prior probability shift, so a calibration temperature optimised on the development distribution does not transfer without adjustment to a more balanced population. This mismatch extends beyond calibration: the class-weighted training and Youden threshold were also derived from the development prior, so their operating characteristics on the near-balanced test cohort should be interpreted with awareness that a shift in prevalence can alter the effective positive and negative predictive values even when sensitivity and specificity remain stable. The post-hoc prior-shift correction (Eq. (11)) reduced both overall and malignant-class test-set ECE, supporting the recommendation that prior-shift correction be applied whenever deployment prevalence differs meaningfully from training prevalence, and that ECE be reported separately per class to distinguish distributional mismatch from genuine miscalibration.

The threshold analysis supports the practical utility of the framework: both the Youden-selected and sensitivity-constrained thresholds produced strong operating characteristics when transferred unchanged from development to the independent test cohort, indicating that the model does not depend on a single arbitrary cutoff. A sensitivity-constrained operating point may be preferred in referral or triage scenarios where reducing missed malignant cases is prioritised over maximising specificity. These findings align with recent work emphasising that calibration and trustworthy probability estimation should be treated as distinct requirements rather than assumed consequences of strong discrimination^17,19^. Relative to the established clinical standard, RCAF substantially exceeded TIRADS-based discrimination on this cohort, principally through higher specificity; this comparison is, however, limited to the ThyroidXL benchmark, and the relative advantage may vary across cohorts with different TIRADS calibration practices and malignancy prevalence profiles.

The high absolute performance on the ThyroidXL test set should be interpreted with caution and is attributable to a combination of dataset-level and methodological factors: (i) the test cohort is pathology-validated and drawn from a standardised, single-source acquisition protocol, which reduces acquisition variability, label noise, and scanner heterogeneity relative to real multi-center deployments; (ii) the near-balanced class distribution of the test cohort is more favourable for ROC-AUC as a ranking metric than the highly imbalanced distributions typical of population screening settings; (iii) patient-level aggregation over multiple frames reduces the influence of low-quality individual frames and may yield higher discrimination than single-image evaluation; and (iv) the TN5000 domain-adapted experiment, in which performance decreased under genuine distribution shift, confirms that the absolute ThyroidXL numbers reflect benchmark-specific conditions rather than an estimate of performance under independent acquisition variability. The marginal excess of the independent-test ROC-AUC over the OOF development mean is consistent with the same effect, since a near-balanced test distribution can improve ROC-AUC as a ranking metric without any change in model calibration or discrimination capacity. The bootstrapped confidence intervals confirm statistical robustness within the ThyroidXL test cohort, but the absolute numbers should be interpreted in the context of this benchmark. The reduction in false positives relative to the lesion-only baseline, visible in the confusion-matrix comparison, is clinically relevant because it suggests RCAF may reduce unnecessary benign-case alarm burden while preserving strong malignant-case detection.

From a methodological perspective, the strict grouped patient-level protocol is a key strength. All model development, calibration fitting, and threshold selection were performed without contamination from the official test set. This is especially important in thyroid ultrasound, where multiple frames from the same examination may share patient-specific appearance patterns; enforcing patient-level separation throughout development and evaluation provides a more realistic estimate of generalisation and a fairer comparison across competing patient-level methods. This emphasis aligns with recent medical AI literature stressing that evaluation design, data quality, and real-world generalisation remain central to the trustworthiness of clinical AI systems^18,19,32^.

## Limitations and future work

This study has several limitations. First, although the study includes external domain-adapted validation on TN5000, broader prospective multi-center validation across independent hospitals, scanners, and acquisition protocols remains necessary before clinical deployment. Second, the framework assumes the availability of segmentation masks for lesion-guided input construction; in a clinical workflow these would need to be generated by a radiologist at acquisition time or by an automated segmentation model applied upstream of the classifier. Although existing thyroid nodule segmentation networks such as that of Dong et al.^14^ perform strongly on comparable data, the mask quality sensitivity analysis (Sect. “Mask quality sensitivity”) shows that RCAF remains competitive under minor-to-moderate segmentation errors while complete mask removal produces a severe performance collapse, motivating end-to-end evaluation with an automated segmentation pipeline. Third, the present work focused on a specific architecture family and a fixed patient-level bag construction strategy, which may discard information in examinations with more than $T_{\max }$ frames. As preliminary evidence on this design choice, OOF ROC-AUC on the full development cohort ($n=3{,}354$; $0.937 \pm 0.012$) and on the subset of patients with at most two frames ($n=1{,}493$; $0.934 \pm 0.022$) differed by only 0.003, indicating negligible sensitivity to bag completeness under current dataset conditions, where the median bag size is 3 and no patient exceeds 10 frames; datasets with routinely longer examinations should nonetheless validate this explicitly. Finally, although the qualitative visualisations and gate-behaviour analysis provide supportive interpretability evidence, they do not by themselves establish causal explanations of model behaviour. These limitations are consistent with broader concerns in medical imaging AI regarding distribution shift, data quality, explainability, and clinical deployment robustness^18,19,33,34^.

Several promising directions follow from these limitations. First, the RCAF encoder is backbone-agnostic by construction, and replacing ResNet50 with ConvNeXt, EfficientNet-V2, Swin Transformer, or thyroid-specific medical foundation models is a natural extension. Second, RCAF is an imaging-only framework by design; incorporating structured clinical metadata such as age, sex, TIRADS score, and laboratory findings via late fusion or cross-modal attention represents a promising multimodal extension. Third, joint multi-task learning in which segmentation and malignancy classification are optimised simultaneously end-to-end is a priority research direction that would remove the dependency on separately provided masks. Fourth, post-hoc attribution methods such as Score-CAM^35^, SHAP, or Integrated Gradients offer a route to deeper interpretability analysis; however, standard implementations of these methods do not transfer directly to the non-standard RCAF pipeline with its dual-branch CNN encoder, gated fusion module, and AttnMIL aggregator, and rigorous adaptation would constitute a dedicated research contribution rather than a straightforward extension. Finally, broader prospective multi-center validation across independently acquired cohorts remains the most important step toward clinical deployment.

## Conclusion

In this work, we presented a patient-level framework for thyroid ultrasound malignancy prediction based on the proposed Region-Aware Context-Aware Fusion (RCAF) model, evaluated under a strict leakage-free protocol. The method combines lesion-focused and context-preserving frame representations through adaptive gated fusion, attention-based bag aggregation, development-only probability calibration, and fixed threshold transfer to an untouched independent test cohort.

Across all experiments, RCAF outperformed stronger fair patient-level baselines and remained stable under post-hoc calibration and clinically relevant threshold selection. Ablation confirmed the contribution of both lesion–context fusion and adaptive gating, shortcut analysis showed that RCAF—unlike naive mask concatenation—does not rely on mask integrity, and mask quality analysis confirmed robustness to realistic segmentation imprecision. External domain-adapted validation on TN5000 provided encouraging evidence of cross-domain adaptability, while the inference profile on mid-range hardware supports practical deployability.

Taken together, these findings indicate that patient-level thyroid ultrasound malignancy prediction benefits from explicitly modelling both lesion morphology and surrounding context within a principled region-aware design. RCAF therefore constitutes a promising foundation for thyroid ultrasound decision-support systems in which reliable patient-level prediction, rather than isolated frame-level scoring, is the central objective, with broader prospective multicentre validation remaining the key next step before clinical deployment.

## Supplementary Information

Supplementary Information.

## Data Availability

The data supporting the findings of this study are derived from the publicly available ThyroidXL dataset, which is cited in the manuscript. Access to the dataset is subject to the access conditions specified by the dataset repository. No new clinical or patient data were collected or generated in this study.

## Code availability

The implementation of the proposed RCAF framework, including the dual-branch encoder, gated fusion module, AttnMIL aggregator, leakage-free grouped cross-validation protocol, temperature scaling calibration, and all evaluation and figure-generation scripts, is publicly available at https://github.com/MennatallahSherif/RCAF_project.
