# Supplementary material for: RCAF for patient-level thyroid ultrasound malignancy prediction under leakage-free evaluation and calibration
Source: Sci Rep. 2026 Jul 16;16:22408. doi: 10.1038/s41598-026-61342-8 (PMC13377050; doi:10.1038/s41598-026-61342-8)
Supplement: Supplementary file 1 — Supplementary Information. [file 41598_2026_61342_MOESM1_ESM.zip › Supplementary/S1_gate_value_distribution.pdf]

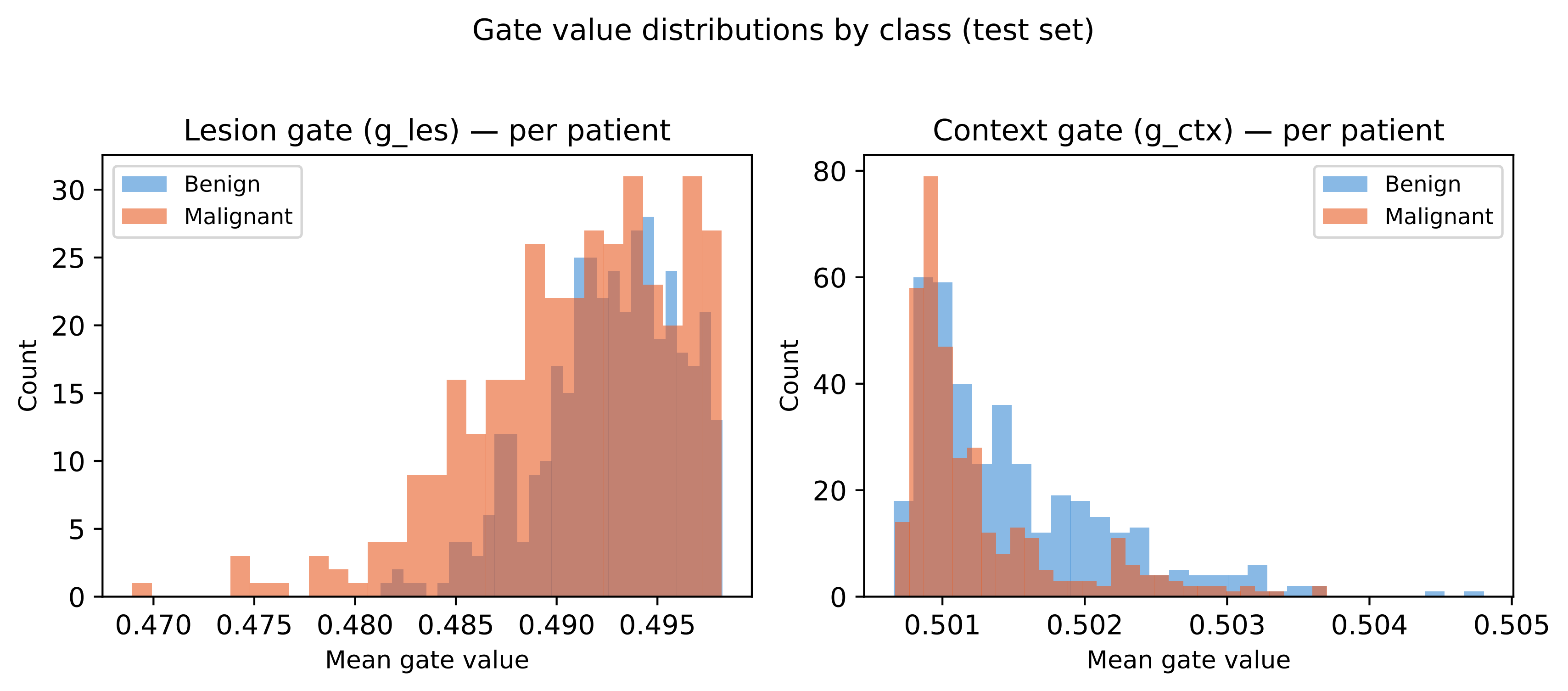
Gate value distributions by class (test set)
Lesion gate (g_les) — per patient
Context gate (g_ctx) — per patient
Benign
Malignant
Count
Mean gate value
0
5
10
15
20
25
30
0.470
0.475
0.480
0.485
0.490
0.495
0
20
40
60
80
0.501
0.502
0.503
0.504
0.505
